# Treatment outcomes and factors affecting treatment outcomes of new patients with tuberculosis in Busan, South Korea: a retrospective study of a citywide registry, 2014–2015

**DOI:** 10.1186/s12879-018-3574-y

**Published:** 2018-12-13

**Authors:** Jeongha Mok, Daeseong An, Seoungjin Kim, Miyoung Lee, Changhoon Kim, Hyunjin Son

**Affiliations:** 1 Department of Internal Medicine, College of Medicine, Pusan National University, Busan, South Korea; 2 Medical Research Institute, Pusan National University Hospital, Busan, South Korea; 3 Busan Center for Infectious Disease Control and Prevention, Pusan National University Hospital, 179 Gudeok-ro, Seo-gu, Busan, 49241 South Korea; 4 Department of Statistics, Pukyong National University, Busan, South Korea; 5 Department of Preventive Medicine, College of Medicine, Pusan National University, Busan, South Korea

**Keywords:** Outcome, South Korea, Tuberculosis

## Abstract

**Background:**

This study investigated the treatment outcomes, and factors affecting the outcomes, of new tuberculosis (TB) patients in Busan, South Korea.

**Methods:**

We retrospectively analysed the citywide TB registry data (collected for the Korean National TB Surveillance System) of new TB patients registered in Busan from January 2014 to December 2015.

**Results:**

A total of 4732 patients were included in this study (mean age, 52.5 ± 19.9 years; 58.4% male). The overall treatment success rate was 83.9% (cured, 20.2%; completed, 63.7%); 8.0% of patients died, and 3.6% were lost to follow-up. In multivariate analyses, a higher rate of loss to follow-up was associated with foreign nationality, registered as TB-positive at least twice, and being in Q4 (fourth quintile) or Q5 (fifth quintile) of the regional deprivation index. Conversely, a lower rate of loss to follow-up was associated with female gender, smear-positive for pulmonary TB (PTB), and the treatment outcome being reported by a public health centre. Higher mortality was associated with old age (≥ 75 years), smear-positive PTB, treatment outcome being reported by the hospital, and being registered as TB-positive twice. Lower mortality was associated with female gender, treatment outcome being reported by a public health centre or clinic, and Q5 of the regional deprivation index.

**Conclusions:**

Treatment outcomes of new TB patients were sub-optimal in Busan. TB control programs should maintain close monitoring and provide greater socioeconomic support to patients at high risk of poor treatment outcomes.

## Background

Despite efforts to eliminate tuberculosis (TB), the incidence and mortality of TB remain high worldwide. In 2016 alone, there were 10.4 million new TB cases globally, and 1.7 million people died from the disease [1]. South Korea is not free from TB, despite having abundant medical resources. In 2017, a total of 36,044 patients in South Korea infected with TB (70.4/100,000 population) were listed on the Korean National TB Surveillance System (NTSS), and 78% of them (28,161 patients; 55.0/100,000 population) were new patients [2]. A total of 2186 patients died from TB in South Korea in 2016 [2]. South Korea has the highest TB incidence and mortality rate of all member countries of the Organization for Economic Co-operation and Development [3], and TB remains a serious public health problem in South Korea.

The “End TB Strategy” of the World Health Organization (WHO) has set a target to increase the TB treatment success rate to 90% by 2025, and to reduce TB deaths by 95% (compared to 2015) by 2035, to end the global TB epidemic [4]. To facilitate these goals and evaluate the effectiveness of the strategy, it is essential to accurately record and report the treatment outcomes TB patients. Treatment outcomes, as measured by a standardised method, are key indicators of national TB program (NTP) effectiveness [5]. In addition to monitoring treatment outcomes, improving treatment success rates is important and depends on identifying vulnerable populations and the risk factors for poor treatment outcomes. This information is important for policymakers in terms of resource planning, prioritisation and distribution.

To achieve the WHO’s TB targets, South Korea has made many changes to its NTP since 2011. The Korea Centers for Disease Control and Prevention (KCDC) outlined the “New 2020 Plan,” which set a target to cut the TB incidence rate in half by 2020 (< 50/100,000 population) [3]. Several programs were implemented to reach this goal, including expanded public-private mix (PPM) collaborations, reinforcement of outbreak investigations, contact investigations and treatment of latent TB infection, forced hospitalisation for non-adherent patients and those with multidrug-resistant (MDR) TB, and making all hospital visits for diagnosis and treatment of TB free-of-charge.

Despite these efforts to improve TB treatment in South Korea, there are limited data regarding recent treatment outcomes of South Korean TB patients and little is known about the effects of the 2011 policy changes. Factors affecting treatment outcomes of South Korean TB patients are not well understood. Most previous studies included only a small number of patients, were conducted in a single institution, or were conducted solely in the private sector [6–8]; therefore, the overall status of TB in South Korea is poorly understood. In the present study, we investigated treatment outcomes of new patients with TB in Busan, which is one of the largest and most TB-prevalent cities in South Korea, using a citywide registry. We also evaluated the factors affecting treatment outcomes of TB patients.

## Methods

### Study design and population

This retrospective cohort study was conducted in Busan, which is the second-largest city in South Korea, with a population of 3.5 million in 2015 (6.8% of the total South Korean population). Notified TB cases in Busan made up 7.5% of all notified TB cases in South Korea. The incidence rates of total and new TB cases in Busan were 87.6 and 68.6/100,000 population in 2015, respectively [9]. The incidence rate of human immunodeficiency virus-positive TB was less than 1/100,000 population in 2015 [10].

All TB patients registered in Busan from January 2014 to December 2015 were screened for inclusion. Among them, new TB patients were included in the analysis. Patients with rifampin-resistant (RR) or MDR-TB were excluded because the long duration of treatment required for these strains made it difficult to confirm treatment outcomes at the time of data collection. Patients with an unknown treatment history, and whose final diagnosis was changed to a disease other than TB, were also excluded. A patient could be registered more than once if they had been transferred from one institution to another during treatment. In total, 1021 (21.6%) of the patients in our cohort were registered at two or more different institutions. We regarded this patient as one case from the beginning of data extraction.

Almost all of the registered TB patients received treatment according to Korean guidelines for TB [11], i.e. a 6-month, self-administered regimen consisting of isoniazid, rifampin, ethambutol and pyrazinamide for 2 months, followed by isoniazid, rifampin, and/or ethambutol for 4 months. An alternative regimen (isoniazid, rifampin, and ethambutol for 9 months) was administered in cases of pyrazinamide intolerance.

The present study protocol was reviewed and approved by the Institutional Review Board of Pusan National University Hospital (IRB approval number: C-1805-001-066). The requirement for obtaining informed consent was waived by the Institutional Review Board of Pusan National University Hospital because this was a retrospective study of routinely collected monitoring data. The dataset was private and provided by the Department of Health and Hygiene of Busan City. Administrative permission for review and analysis of the patient’s record was obtained from the Department of Health and Hygiene of Busan City. The data were de-identified prior to being analysed.

### Data source

TB is a mandatorily notifiable disease in South Korea. All patients diagnosed with TB are registered with the Korean NTSS, which is a web-based registration system launched in 2000 by the KCDC. The citywide registry data used in the present study were a subset of this Korean NTSS data. The Korean NTSS includes the following information: identification number, age, sex, nationality, address, TB case classification (based on history of previous TB treatment), type of TB (pulmonary TB [PTB] or extrapulmonary TB [EPTB]), type of institution that registered the patient initially, type of institution that reported the final treatment outcome, results of acid fast bacilli (AFB) smear and culture, drug susceptibility test results, and treatment outcome.

**Fig. 1:**
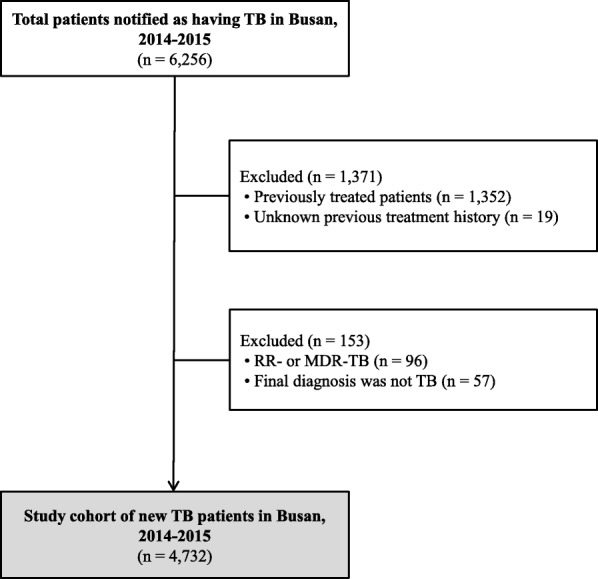
Flowchart of selection of the study participants. MDR, multidrug-resistant; RR, rifampin-resistant; TB, tuberculosis

A flowchart showing the process followed to establish the study cohort of new TB patients registered in Busan in 2014–2015 is presented in Fig. 1. The registry data of TB patients in Busan were downloaded from the Korean NTSS server in November 2016, so the treatment outcomes of our 2014–2015 study cohort included follow-up for at least 11 months after diagnosis. For patients who were registered as TB-positive more than once, we selected the latest treatment outcomes. However, if the latest treatment outcomes were “not evaluated,” we examined the notified raw data of each patient and, if possible, selected treatment outcomes other than “not evaluated.”

### Definitions

All definitions used in the study were in accordance with WHO definitions [5]. Patients were classified into two groups according to their TB treatment history: new patients were those who had never been treated for TB or who had taken anti-TB drugs for < 1 month, and previously treated patients were those who had received anti-TB drugs for ≥1 month. Treatment outcomes were categorised as follows: cured (PTB patient with bacteriologically confirmed TB at the beginning of treatment who was smear- or culture-negative in the last month of treatment and on at least one previous occasion; treatment completed (TB patient who completed treatment without evidence of failure but with no record of being cured); treatment failed (TB patient whose sputum smear or culture was positive at month 5 or later during treatment); died (TB patient who died for any reason before or during the course of treatment); lost to follow-up (TB patient who did not start treatment or whose treatment was interrupted for 2 consecutive months or more); and not evaluated (TB patient to whom no treatment outcome was assigned, including cases that transferred to another institution). We defined treatment success as the sum of cured and treatment-completed cases. In 2015, the KCDC revised the definition of “cured” by eliminating the smear criterion. Thus, in the 2015 cohort, the treatment outcome “cured” was evaluated based only on culture results.

The Korean NTSS does not record socioeconomic data of patients. Therefore, we used a regional deprivation index. The regional deprivation index is used to estimate the socioeconomic status of an area (town-level by address in our study), and is regarded as an indirect indicator of the socioeconomic status of patients in a given area. The index is the sum of the following nine socioeconomic variables: house environment, educational level, social class of head-of-household and proportion of elderly people, single household, household without a car, non-apartment housing, female-headed household, and divorced or separated household. Higher index values indicate more deprivation, with Q5 (fifth quintile) corresponding to the most severe deprivation, and Q1 (first quintile) to the lowest level of deprivation [12].

### Statistical analyses

Continuous variables are expressed as means ± standard deviation and categorical variables as percentages. Continuous and categorical variables were compared using ANOVAs and chi-squared tests, respectively. To evaluate the factors affecting treatment outcomes, multivariate logistic regression was performed. Sensitivity analysis was also performed, in which all patients listed as “not evaluated” were assumed to be “lost to follow-up.” All statistical analyses were performed using SAS software (ver. 9.4; SAS Institute Inc., Cary, NC, USA). Two-tailed *p* values < 0.05 were accepted as statistically significant.

## Results

### Baseline characteristics

A total of 6256 patients were screened during the study period. After application of the criteria outlined above, 4732 patients were included in the study cohort (Fig. 1). Baseline characteristics of the patients are summarised in Table 1. The mean age was 52.5 ± 19.9 years, and 58.4% were male; 1.6% (*n* = 78) of total patients were of foreign nationality. PTB was the most common type of TB (*n* = 3771, 79.7%), and 38.8% of the PTB patients showed positive smear results. Regarding the notifying institution, the private sector institutions notified over 90% of the study cohort (*n* = 4262). There was no significant difference in baseline characteristics between 2014 and 2015.

**Table 1:** Baseline characteristics of the study cohort

	*n* = 4732
Year notified as having tuberculosis	
2014	2455 (51.9)
2015	2277 (48.1)
Age, years	
0–9	11 (0.2)
10–19	196 (4.1)
20–44	1451 (30.7)
45–59	1190 (25.1)
60–74	1117 (23.6)
≥ 75	767 (16.2)
Sex	
Male	2765 (58.4)
Female	1967 (41.6)
Nationality	
Korean	4654 (98.4)
Foreign	78 (1.6)
Type of tuberculosis	
Pulmonary	3771 (79.7)
Smear positive	1464 (30.9)
Smear negative	2307 (48.8)
Extrapulmonary	961 (20.3)
Type of institution initially registering patient	
Public health centre	470 (9.9)
Private	4262 (90.1)
PPM participating general hospital	2534 (53.6)
PPM non-participating general hospital	1026 (21.7)
Hospital	478 (10.1)
Clinic	224 (4.7)
Number of registrations as TB-positive	
1	3711 (78.4)
2	849 (17.9)
≥ 3	172 (3.6)
Regional deprivation index (quintile)^a^	
Q1	1135 (24.4)
Q2	1237 (26.6)
Q3	1213 (26.0)
Q4	729 (15.7)
Q5	344 (7.4)

*PPM* public-private mix, *TB* tuberculosis

Data are presented as numbers (%)

^a^*n* = 4658 (variables of 74 patients were missing)

### Treatment outcomes

Treatment outcomes are listed in Table 2. The overall treatment success rate was 83.9% (cured, 20.2%; completed, 63.7%) and 8.0 and 3.6% of patients died and were lost to follow-up, respectively. Treatment outcomes of “completed” and “not evaluated” were more common in 2015 than in 2014.

**Table 2:** Treatment outcomes of the study cohort by year

	Total (*n* = 4732)	2014 (*n* = 2455)	2015 (*n* = 2277)	*P* ^a^
Cured	955 (20.2)	603 (24.6)	352 (15.5)	< 0.001
Completed	3015 (63.7)	1501 (61.1)	1514 (66.5)	< 0.001
Failed	4 (0.1)	3 (0.1)	1 (0.0)	0.626
Lost to follow-up	171 (3.6)	88 (3.6)	83 (3.6)	0.911
Died	379 (8.0)	192 (7.8)	187 (8.2)	0.620
Not evaluated	208 (4.4)	68 (2.8)	140 (6.1)	< 0.001

Data are presented as numbers (%)

^a^Comparison between 2014 and 2015

Table 3 shows the treatment outcomes according to patient characteristics. Higher treatment success rates were seen in patients who were younger (especially those aged 10–19 years), female, of Korean nationality, showed smear-negative PTB or EPTB, had treatment outcomes reported by a public health centre, or were registered as TB-positive once. The rate of loss to follow-up was higher in patients who were male, foreign nationals, diagnosed with EPTB, or had their treatment outcome reported by institutions other than a public health centre. Mortality was higher among patients who were older (≥ 75 years), male, of Korean nationality, diagnosed with smear-positive PTB, had their treatment outcome reported by the hospital, or were registered as TB-positive at least twice.

**Table 3:** Treatment outcomes of the study cohort according to baseline characteristics

	Total^a^ (*n* = 4732)	Success (*n* = 3970)	*P*	Lost to follow-up^b^ (*n* = 379)	*P*	Died (*n* = 379)	*P*
Age, years			< 0.001		0.229		< 0.001
0–9	11	10 (90.9)		0 (0.0)		1 (9.1)	
10–19	196	187 (95.4)		9 (4.6)		0 (0.0)	
20–44	1451	1331 (91.7)		107 (7.4)		10 (0.7)	
45–59	1190	1042 (87.6)		106 (8.9)		41 (3.5)	
60–74	1117	893 (80.0)		97 (8.7)		127 (11.4)	
≥ 75	767	507 (66.1)		60 (7.8)		200 (26.1)	
Sex			< 0.001		< 0.001		0.002
Male	2765	2249 (81.3)		264 (9.5)		250 (9.0)	
Female	1967	1721 (87.5)		115 (5.8)		129 (6.6)	
Nationality			< 0.001		< 0.001		0.009
Korean	4654	3919 (84.2)		352 (7.6)		379 (8.1)	
Foreign	78	51 (65.4)		27 (34.6)		0 (0.0)	
Type of tuberculosis			0.011		0.018		< 0.001
Pulmonary, smear positive	1464	1193 (81.5)		98 (6.7)		172 (11.7)	
Pulmonary, smear negative	2307	1961 (85.0)		186 (8.1)		158 (6.8)	
Extrapulmonary	961	816 (84.9)		95 (9.9)		49 (5.1)	
Type of institution^c^			< 0.001		0.043		< 0.001
Public health centre	551	519 (94.2)		27 (4.9)		4 (0.7)	
General hospital^d^	3489	2910 (83.4)		298 (8.5)		278 (8.0)	
PPM participating	2542	2117 (83.3)		212 (8.3)		210 (8.3)	
PPM non-participating	947	793 (83.7)		86 (9.1)		68 (7.2)	
Hospital	545	409 (75.0)		40 (7.3)		96 (17.6)	
Clinic	147	132 (89.8)		14 (9.5)		1 (0.7)	
Number of registrations as TB-positive			< 0.001		0.148		< 0.001
1	3711	3169 (85.4)		284 (7.7)		254 (6.8)	
2	849	669 (78.8)		76 (9.0)		104 (12.2)	
≥ 3	172	132 (76.7)		19 (11.0)		21 (12.2)	
Regional deprivation index (quintile)^e^			0.556		0.820		0.201
Q1	1135	85.4 ± 5.6		3.1 ± 2.8		8.0 ± 3.7	
Q2	1237	83.7 ± 6.1		2.9 ± 2.0		8.3 ± 5.5	
Q3	1213	83.1 ± 6.5		3.9 ± 3.1		8.6 ± 5.6	
Q4	729	80.7 ± 12.4		3.7 ± 4.0		10.6 ± 8.8	
Q5	344	83.8 ± 11.6		4.1 ± 6.5		5.5 ± 6.0	

*PPM* public-private mix, *TB* tuberculosis

Data are presented as numbers (%) or means ± standard deviations

^a^Including four patients whose treatment failed

^b^Including 208 patients whose treatment outcome was not evaluated

^c^Type of institution that reported the final treatment outcome

^d^There was no significant difference between PPM participating and non-participating general hospitals in rates of treatment success, loss to follow-up, or death

^e^*n* = 4658 (data missing for 74 patients)

### Factors affecting treatment outcomes

Table 4 shows the results of multivariate analysis of factors affecting treatment outcomes. Higher treatment success rates were seen in patients who were female or whose treatment outcome was reported by a public health centre. Conversely, lower treatment success rates were seen in patients who were registered in 2015, were ≥ 45 years old, were foreign nationals, were registered as TB-positive twice, or were classified as Q4 in the regional deprivation index.

**Table 4:** Multivariate analysis of factors affecting treatment outcomes

	Success		Lost to follow-up^a^		Died	
	OR	95% CI	OR	95% CI	OR	95% CI
Year notified as having tuberculosis						
2014	1.00	reference	1.00	reference	1.00	reference
2015	0.81	0.69–0.95	1.53	1.23–1.90	0.95	0.76–1.20
Age, years						
10–19	1.00	reference	1.00	reference	–	
0–9	0.51	0.06–4.53	–		0.50	0.06–4.11
20–44	0.66	0.33–1.34	1.42	0.69–2.90	0.02	0.01–0.05
45–59	0.41	0.20–0.83	1.91	0.94–3.91	0.10	0.07–0.14
60–74	0.24	0.12–0.48	1.80	0.88–3.69	0.35	0.27–0.46
≥ 75	0.11	0.06–0.22	1.72	0.82–3.60	1.00	reference
Sex						
Male	1.00	reference	1.00	reference	1.00	reference
Female	1.75	1.46–2.08	0.59	0.47–0.75	0.58	0.46–0.75
Nationality						
Korean	1.00	reference	1.00	reference	–	
Foreign	0.15	0.09–0.24	8.48	5.06–14.21	–	
Type of tuberculosis						
Extrapulmonary	1.00	reference	1.00	reference	1.00	reference
Pulmonary, smear negative	0.91	0.73–1.14	0.79	0.60–1.03	1.72	1.21–2.44
Pulmonary, smear positive	0.83	0.65–1.04	0.62	0.46–0.84	2.54	1.79–3.62
Type of institution^b^						
PPM participating general hospital	1.00	reference	1.00	reference	1.00	reference
PPM non-participating general hospital	1.09	0.88–1.35	1.05	0.80–1.37	0.83	0.61–1.13
Hospital	0.89	0.70–1.14	0.75	0.52–1.09	1.46	1.07–1.98
Clinic	1.61	0.92–2.83	1.26	0.70–2.25	0.08	0.01–0.57
Public health centre	3.32	2.24–4.91	0.52	0.34–0.80	0.10	0.04–0.26
Number of registrations as TB-positive						
1	1.00	reference	1.00	reference	1.00	reference
2	0.67	0.54–0.83	1.39	1.05–1.84	1.47	1.10–2.00
≥ 3	0.72	0.48–1.07	1.90	1.14–3.16	0.97	0.57–1.66
Regional deprivation index (quintile)						
Q1	1.00	reference	1.00	reference	1.00	reference
Q2	0.93	0.73–1.17	1.09	0.79–1.50	1.01	0.73–1.40
Q3	0.99	0.78–1.25	1.05	0.77–1.44	0.98	0.70–1.35
Q4	0.73	0.56–0.95	1.53	1.09–2.15	1.13	0.79–1.62
Q5	1.01	0.71–1.43	1.54	1.01–2.35	0.56	0.32–0.96

*CI* confidence interval, *OR* odds ratio, *PPM* public-private mix, *TB* tuberculosis

^a^Including 208 patients whose treatment outcome was not evaluated

^b^Type of institution that reported final treatment outcome

Higher rates of loss to follow-up were seen in patients who were registered in 2015, were foreign nationals, were registered as TB-positive at least twice, or who were classified as Q4 or Q5 in the regional deprivation index. Female gender, smear-positive PTB, and treatment outcome reported by a public health centre were protective against loss to follow-up.

Higher mortality was associated with older age (≥ 75 years), diagnosis of PTB (especially smear-positive PTB), treatment outcome reported by a hospital, and two registrations as TB-positive. On the other hand, lower mortality was associated with female gender, treatment outcome being was reported by a public health centre or clinic, and classification of Q5 in the regional deprivation index.

## Discussion

To our knowledge, this is the first large-scale study to investigate the treatment outcomes, and factors affecting outcomes, of new patients with TB in Busan, South Korea. A major strength of our study was that it included a large cohort of TB patients, drawn from the population of a major city. Another strength is that we were able to include patients who transferred to other hospitals within the city, by using patient identification numbers to determine treatment outcomes. The Korean NTSS successfully registered 94.0% of all TB cases in 2014 [13], so it is likely that our results are representative of the overall TB status of Busan.

During the study period, the treatment success rate of new TB patients in Busan was 83.9%, which was similar to that of South Korea as a whole (84.4% for 2014 and 83.8% for 2015) [14]. However, neither rate met the 90% target set by the WHO. This sub-optimal treatment success rate was mainly due to high mortality (8.0%). One of the main risk factors for death of TB patients is old age. A lower in treatment success rate due to the high incidence of death of elderly TB patients is typical of both low- and high-TB incidence countries [15–17]. Several factors are associated with death in elderly TB patients. Besides aging per se, high mortality in elderly TB patients may be due to age-related comorbidities, reduced immune function, missed diagnosis and delayed treatment initiation due to atypical presentation of TB, and difficulties in accessing healthcare services [18–21]. In our cohort, elderly TB patients (≥ 60 years old) accounted for nearly 40% of all of the patients and, not surprisingly, old age was an independent risk factor for death. The number of elderly TB patients is expected to increase as life expectancy continues to improve, which presents major challenges to TB control in South Korea [22]. In addition to old age, male gender and PTB (especially smear-positive PTB) were also independently associated with high mortality in our study, which is consistent with previous reports [17, 23]. Higher death rates in male patients may be due to behaviours such as alcohol consumption, smoking, and non-adherence to treatment [24–27]. Patients with smear-positive PTB may have advanced disease. Treatment outcomes being reported by a hospital was another risk factor for death in our study, but this could be due to selection bias: a considerable number of TB patients who have chronic critical illness and limited options for treatment are transferred to such hospitals in the post-infectious stage, likely leading to higher acuity of hospital patients. Unexpectedly, Q5 of the regional deprivation index was associated with lower mortality. However, this was attributable to the higher rate of loss to follow-up in the Q5 group; we estimate that a significant proportion of the patients lost to follow-up in the Q5 group were indeed dead.

To reduce death from TB, interventions for elderly patients should be prioritised. Elderly patients are more likely to show an atypical presentation of TB, so actively searching for cases would facilitate early diagnosis and timely treatment, which would in turn likely reduce mortality [28, 29]. The standard sputum AFB smear has low sensitivity in elderly patients, making TB detection more difficult, but molecular tests such as the Xpert MTB/RIF assay could overcome this problem [30]. Administrative support, such as financial support and help with transportation, could improve access to healthcare services for vulnerable elderly patients. TB-related deaths are common in the intensive treatment phase [7, 16], so intensive monitoring early in this treatment period should be considered not only for elderly patients, but also for patients with other risk factors for death (e.g. male, smear-positive PTB, non-adherence to treatment).

The number of elderly TB patients is expected to increase in the future, and efforts to reduce mortality of elderly patients may be stymied by the inherent demographic characteristics of this population. In addition, all new patients with TB had low rates of treatment failure which would be difficult to decrease further. Therefore, to achieve WHO targets, a paradigm shift in TB control strategies may be necessary to reduce non-adherence to treatment among patients in Busan. Non-adherence results in serious problems not only for the patients themselves (including development of complications, acquired drug resistance, and death), but also for communities in terms of the spread of infection [31]. In our study, male gender, foreign nationality, smear-negative PTB and EPTB, and treatment outcome reported by institutions other than public health centres were risk factors for loss to follow-up, which was consistent with previous studies [32–35]. The number of TB patients of foreign nationality has increased in South Korea (from 849 patients in 2010 to 2045 in 2017) [2]. The higher rate of loss to follow-up in these foreign national populations may be due to difficulties in accessing healthcare services due to legal, employment, economic, or language problems [33]. The higher rate of loss to follow-up in cases of EPTB may be due to excessively long treatment durations, lack of motivation due to lower symptom severity compared to PTB, or reduced attention from TB control programs due to lower infectivity [36, 37].

To improve patient compliance, PPM collaborations should be expanded throughout South Korea, as well as in Busan specifically, to enhance TB control in the private sector. A previous South Korean study showed that PPM collaborations decreased the rate of loss to follow-up in the private sector [38]. For non-adherent patients of foreign nationality, international collaborations to ensure continuity of treatment after departure could be useful, in addition to administrative support to improve access to healthcare services [39]. Patient-centred care is also important for reducing non-adherence to treatment. This may include providing sufficient information on TB to patients, improving communication between patients and healthcare providers, and providing social support to help overcome the stigma of TB. Although studies of the efficacy of directly observed therapy (DOT) have reported inconclusive results, DOT may be considered for non-compliant patients [3, 35, 40, 41].

There were several limitations to this study. First, socioeconomic parameters that could affect patient treatment outcomes were not recorded (e.g. smoking status, alcohol consumption, level of education, degree of employment, and economic status). We used a regional deprivation index as an indirect indicator of socioeconomic status, but it showed inconsistent associations with treatment outcomes. Because the regional deprivation index is based on the data of a given area (town level), not a patient, it might not reflect the socioeconomic status of the individual patient. Also, Busan is one of the most highly developed metropolitan cities in South Korea, so differences in regional deprivation might not be particularly significant. Further, socioeconomic differences may be diluted by free-of-charge hospital visits for TB and relatively easy access to healthcare services for all patients in Busan. Second, we did not include previously treated patients, or those with RR- or MDR-TB in the analyses. These patients are well known as high-risk groups for poor treatment outcomes. Further investigations are needed on these vulnerable populations. Finally, we could not distinguish between TB-related and non-TB related deaths. Although the WHO defines TB death as that occurring during TB treatment, irrespective of cause, knowing the actual cause of death may help to improve TB control programs.

## Conclusion

Treatment outcomes of new TB patients were found to be sub-optimal in Busan, South Korea. To reduce mortality and loss to follow-up rates, and therefor increase the treatment success rate, TB control programs should involve close monitoring and provide more socioeconomic support to patients at high risk for poor treatment outcomes.

## Abbreviations

AFB: Acid-fast bacilli
DOT: Directly observed therapy
EPTB: Extrapulmonary tuberculosis
KCDC: Korea Centers for Disease Control and Prevention
MDR: Multidrug-resistant
NTP: National tuberculosis program
NTSS: National TB Surveillance System
PPM: Public-private mix
PTB: Pulmonary tuberculosis
RR: Rifampin-resistant
TB: Tuberculosis
WHO: World Health Organization
